# Managing the economic challenges in the treatment of heart failure

**DOI:** 10.1186/s12872-021-02408-5

**Published:** 2021-12-25

**Authors:** Ileana L. Piña, Larry A. Allen, Nihar R. Desai

**Affiliations:** 1 Wayne State University, Detroit, USA; 2 Central Michigan University, Mount Pleasant, MI USA; 3 Division of Cardiology, University of Colorado School of Medicine, Aurora, CO USA; 4 Cardiovascular Medicine, Yale University School of Medicine, New Haven, CT USA; 5 Wayne State University, 2627 Fairmount Boulevard, Cleveland Heights, OH 44106 USA

**Keywords:** BPCI-advanced, Heart failure, Cost-effectiveness, Telehealth, Payment models, Value

## Abstract

**Background:**

Treatment of heart failure is complex and inherently challenging. Patients traverse multiple practice settings as inpatients and outpatients, often resulting in fragmented care. The Center for Medicare and Medicaid Services is implementing payment programs that reward delivery of high-quality, cost-effective care, and one of the newer programs, the Bundled Payment for Care Improvement Advanced program, attempts to improve the coordination of care across practices for a hospitalization episode and post-acute care. The quality and cost of care contribute to its value, but value may be defined in different ways by different entities.

**Conclusions:**

The rapidly changing world of digital health may contribute to or detract from the quality and cost of care. Health systems, payers, and patients are all grappling with these issues, which were reviewed at a symposium at the Heart Failure Society of America conference in Philadelphia, Pennsylvania on September 14, 2019. This article constitutes the proceedings from that symposium.

## Background and program overview

### Ileana L. Piña, symposium chair

The care of patients with heart failure (HF) continues to be complex, fragmented, and costly. Large health systems and private practices, academic and community centers, policymakers and those participating in various payment models, are all trying to navigate this landscape. Central is the patient, who needs high-quality, coordinated care involving regular interactions with the health care system. Digital-health has the potential to increase quality, reduce costs, and improve coordination of care, but brings other challenges. The Center for Medicare and Medicaid Services (CMS) is addressing challenges presented by complex conditions such as HF through the Bundled Payment for Care Improvement (BPCI) Advanced program, which intends to streamline coordinated care episodes that are patient-centered, and improve the patient experience and outcomes, while reducing costs.

The 4th annual symposium titled “Managing the Economic Challenges in the Treatment of Heart Failure” explored the BPCI Advanced program, how value is interpreted in the context of HF care, and the role of remote medicine in managing HF. The faculty were Steven A. Farmer, MD, PhD, who introduced the BPCI Advanced program goals and structure; Nihar Desai, MD, MPH, who described the evolution of payment models; Paul Heidenreich, MD, MS, who discussed the concept of value in the treatment of HF; and, Larry Allen, MD, who provided perspectives on the impact of remote medicine on HF outcomes and cost.

Participants were surveyed about their experiences and views on these issues throughout the program: Physicians (20%), advanced practice professionals (17%), researchers (16%), pharmacists (7%), nurses (7%), administrators (3%), and others (30%). Practice settings were inpatient/outpatient (32%), outpatient (15%), inpatient (4%), and other (49%, excluding skilled nursing facilities [SNF] and long-term nursing facilities). Most practiced in either the Northeast (37%) or West (30%), and the rest split evenly between the South and Midwest. Thirteen percent knew their institution was participating in BPCI Advanced, and 43% were unsure.

**Fig. 1:**
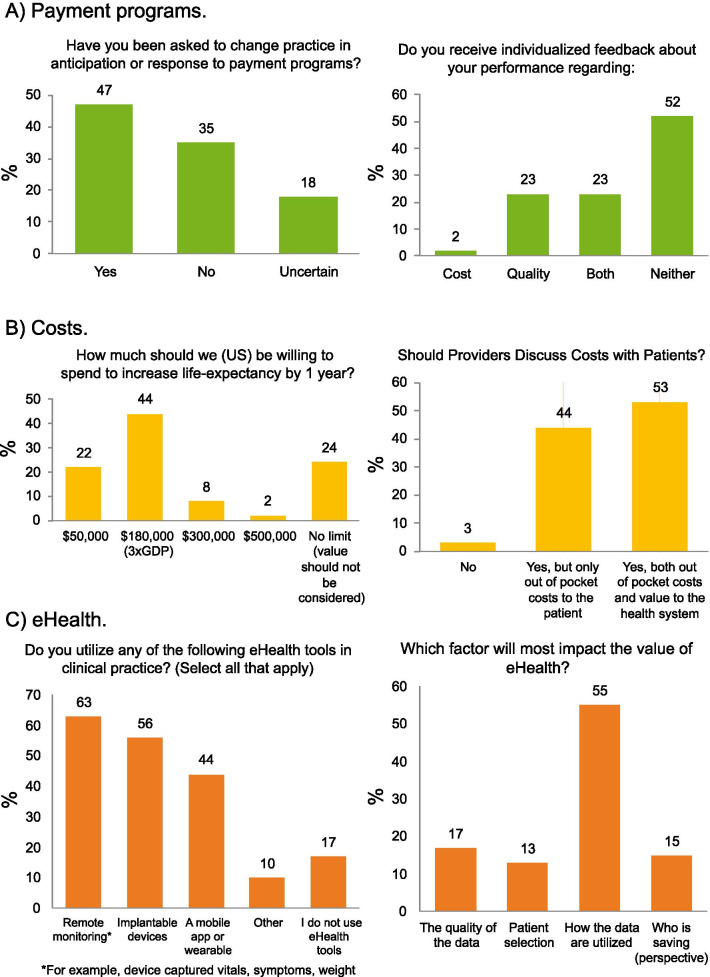
Participant responses to questions by topic. **a** Payment programs, **b** costs; **c** eHealth

Figure 1 shows participant responses to questions on (*A*) the impact of payment programs on practice, (*B*) perceptions around costs, and (*C*) perspectives on eHealth. Briefly, 47% had been asked to change practice due to a payment program (18% were uncertain), but 52% had not received feedback about either cost or quality; 44% felt that $180,000 was an appropriate spend to increase life expectancy by 1 year (24% felt no cost limit), and all but 3% felt that cost should be discussed with patients. All but 17% used at least one eHealth tool in their practice, and 55% believed that how the data would be used would impact the value of eHealth. These responses open the door to new opportunities for health systems to involve and inform providers in their costs and payment model decisions.

The symposium was supported by Cytokinetics as an unrestricted educational grant. The faculty had complete freedom to set the agenda and prepare the content. The faculty have prepared the following proceedings based on their presentations at the symposium.

## Payment models for heart failure: insights from the CMS bundled pilot program

### Nihar R. Desai

**Fig. 2:**
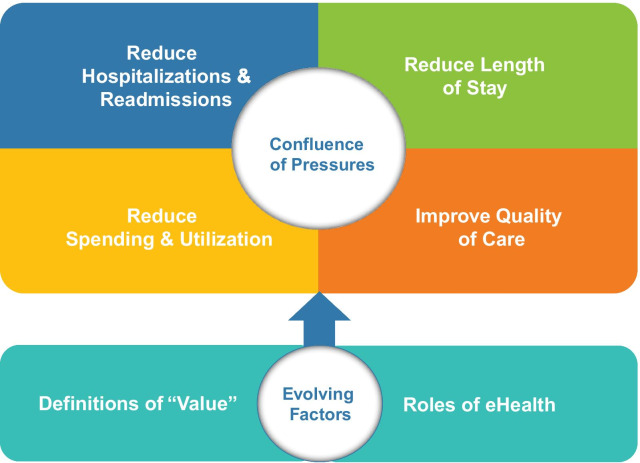
Policy environment and HF care

Medicine is experiencing a remarkable period of scientific, technological, and therapeutic advances concurrent with an extraordinary period of health care policy, care delivery, and payment reform catalyzed by substantial variation in health care quality and cost. Within this complex environment, policymakers have sought to achieve 4 key objectives (Fig. 2): (a) reduce hospitalizations and readmissions; (b) reduce length of stay; (c) improve quality; and (d) reduce spending, especially low-value services. Policymakers have relatively few tools at their disposal to improve quality and address cost growth. One tool is to change the payment system; consequently, the payment model is rapidly evolving away from a fee-for-service system, where reimbursement is linked to the volume of services delivered, toward a value-based system, where payment is linked, at least in part, to the quality and efficiency of care and outcomes achieved.

Several value-based programs have been developed, and include the Hospital Readmission Reduction Program and the Hospital Value-Based Purchasing Program and the Merit-Based Incentive Payment System for ambulatory care as examples of pay-for-performance programs [1]. These programs focus on specific measures and quality domains, but are ultimately narrowly focused. Empirical analyses of the impact of Hospital Readmission Reduction Program and Hospital Value-Based Purchasing Program on quality and outcomes have yielded mixed results. At the other end of the spectrum are Accountable Care Organizations (ACOs), which assume responsibility and financial risk to provide population-based care [1]. ACOs are rewarded financially when they improve care coordination, quality outcomes, and efficiency. Early experience with ACOs has shown a modest benefit with cost reductions, but not necessarily improved care quality [2].

Bundled-payment programs are intermediate options between the pay-for-performance and ACO programs in that they are anchored to a clinical episode or condition, and incorporate incentives for providing high-quality, cost-efficient care. Bundled payments are intuitively appealing, particularly to hospitals and health systems, and based on the substantial variation in outcomes and cost that have been reported. More specifically, in work led by the Yale Center for Outcomes Research and Evaluation, the data for an index hospitalization and subsequent 30-day episode of care show substantial variation between hospitals in how much spending and utilization occurs for the care of HF patients: $11,000–$22,000, or about a twofold variation in spending [3]. About 60% of spending is related to the index hospitalization, and about 40% to post-acute care with the biggest drivers of post-acute spending being readmissions and SNFs.

Another interesting and policy-relevant question is whether there is a relationship between bundling and improved outcomes. A group of Yale investigators examined the relationship between the hospital-level, 30-day risk-standardized payment and 30-day risk-standardized mortality rates for HF [4]. Sampling 4641 hospitals with 960,960 hospitalizations for mortality and 903,721 hospitalizations for payment in the U.S., there was a statistically significant but weak association between risk-standardized mortality rate and risk-standardized payment (correlation coefficient: − 0.21 [95% confidence interval (CI): − 0.24 to − 0.18]). Thus, bundles may provide an opportunity to improve the value of care in HF management.

The BPCI Advanced program was launched in January 2018 to improve quality and reduce cost of care for Medicare beneficiaries [5]. The overarching goals were to redesign care; engage healthcare providers, patients, and caregivers; provide data analysis and feedback; and impose accountability for the quality and cost of care. HF is one of the 29 inpatient clinical episodes defined in the BPCI Advanced model for which physician group practices and hospitals may voluntarily participate in the program. Each clinical episode is triggered by an index hospitalization and extends from the inpatient stay to 90 days post-discharge. CMS provides fee-for-service payments for individual health care services during the episode, then reconciles the overall cost against a predetermined target price. If the total cost during that 90-day period is below the target price, the participant may retain the difference; if the total cost is above the target price, the participant must reimburse CMS for the difference. Rewards and penalties under the model are adjusted for quality performance. High-level strategies for succeeding in the program are to ensure efficient, high-quality inpatient care; reduce rehospitalizations; reduce unnecessary post-acute care; optimize the efficiency of post-acute care; and perform well on prespecified outcome measures.

Several critical success factors for participation in BPCI Advanced have been identified [6]. Meaningful engagement of clinical providers is crucial for success. Sufficient episode volume is necessary to present an adequate savings opportunity and minimize chance variation in performance. “Actionable costs,” defined as those that can be affected by changes in care delivery, are a management focus in the model. For episodes like HF, patient education and engagement and improved care coordination are crucial to avoid unnecessary post-acute care expenses, duplicative testing, and readmissions.

The Yale Health System had to build the capacity to participate in BPCI Advanced and had to define quality and financial opportunity through systematic and rigorous analysis. This required engagement in and commitment to a data-driven decision-making process. The ability to integrate clinical, operational, and financial data was required to make the kinds of strategic decisions and operational improvements needed for success in a program like BPCI Advanced. Detailed analysis of historical expenditures for HF, including the index stay, SNFs, and readmissions, was important to guide the process and decisions. “Actionable spend” was defined to identify opportunities where effort and investment would yield rewards, with the target price set by Medicare as the ultimate benchmark.

The strategy employed at the Yale Health System included internal (within the system) and external (outside the system) components. Internally, the focus was on clinical triage to the right bed and the right team, consistent use of guideline-directed medical therapy (GDMT), interventions to reduce readmission, and managing length of stay. Externally, the focus was on reducing rehospitalizations, optimizing post-acute care utilization, reducing SNF length of stay, and increasing home health capacity. Partnership building for the post-acute care not provided by the Yale New Haven Health System was critical to the success of the program, and a care integration specialist was hired to help better coordinate care and facilitate communication with post-acute care providers. This strategy required building new data analytic capacity, and establishing partnerships to supplement internal analytic expertise. It also required developing HF patient dashboards that could update in real time and provide information on care quality and use of GDMT in real time. Specific targets and standards for internal, SNF, and home health sectors had to be established. Collectively, capacity building, organizational alignment and readiness, strategy and operations teams for inpatient and post-acute care, and financial risk sharing, have primed the Yale Health System to “play the long game” and be successful in the rapidly evolving landscape of alternative payment models.

## Value of new therapeutic modalities: what are the appropriate tools/metrics?

### Ileana Piña; based on the presentation by Paul A. Heidenreich, MD

**Fig. 3:**
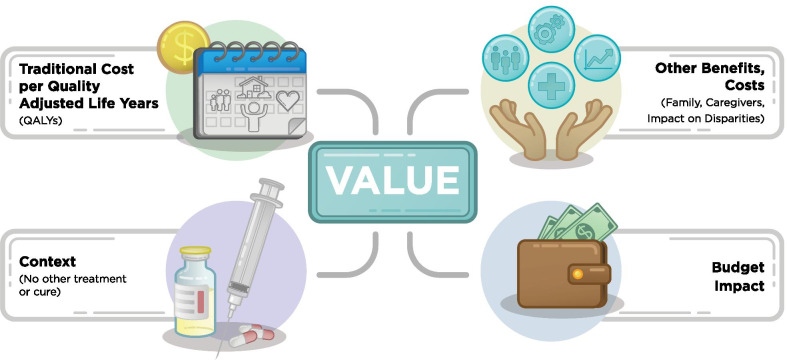
Considerations in defining value

When considering the value of a health care intervention, cost is just one aspect. Traditionally, economists and the greater health care community, have used cost per quality-adjusted life year (QALY) as a measure of value. The Institute for Clinical and Economic Review (ICER) suggests other measures [7], including family or caregiver impact, effects on disparities among patient groups, and the context of available treatment options (Fig. 3). For example, interventions that increase patient independence or ability to return to work, decrease overall treatment burden (e.g., oral versus infused treatment), or reduce the impact of the disease on caregivers have a substantial impact the overall value of an intervention. Similarly, an intervention that differentially benefits an underserved patient population have great value from a patient and societal perspective. ICER acknowledges that these elements of value that are important to patients are poorly captured in clinical trials [7]. Furthermore, how should we assign value to a curative treatment, while considering its impact on the overall health-care budget? If a large percentage of the population were affected with a condition, a cost-effective treatment as defined by QALY may bankrupt the health care system. Nonetheless, the cost per QALY will likely be how most continue to assess value.

Once a measure of value is determined, we need a benchmark for good value. The World Health Organization (WHO) has suggested that interventions with ratios below 1× gross domestic product (GDP) per capita per QALY would generally be considered affordable and cost-effective [8]. In the US, this is between $60,000 and $65,000 per life year gained [9]. WHO has also suggested a benchmark of 3× GDP per capita as an upper limit of cost-effectiveness in any country. Therefore, in the US, between $60,000 and $180,000 per QALY gained would be considered cost-effective, and above this threshold would be economically undesirable. Ideally, all interventions would be both low cost and highly effective. Realistically, efficacy is variable, and therapies, particularly new ones, are often high cost. Society will need to determine the threshold at which a therapy’s cost for the value (effectiveness) is determined to be acceptable or unacceptable.

Cost-effectiveness and value of treatment for patients in a framework of willingness-to-pay typically correlates with an individual’s salary. Twice the median per-capita salary has often been quoted as acceptable. In the US, this would be approximately $120,000 per QALY, consistent with the WHO suggestion. Other perspectives include those of society, payers, hospitals, and providers. Even within these stakeholder categories, views are not homogeneous and can even be conflicting. For example, the American College of Cardiology and American Heart Association (ACC/AHA) adopted the WHO’s perspective. If a health intervention exceeds 3× GDP per capita (or $180,000) per QALY, it is not considered cost-effective [8]. The recent guidelines on cholesterol management included a statement on the value of proprotein convertase subtilisin/kexin type 9 (PCSK9) inhibitors; the price of these medications at the time was > $180,000 per QALY, and the ACC/AHA considered them “low value” unless restricted to the highest-risk populations [10].

According to several cost-effectiveness analyses [11–13], most treatments in the HF landscape, including angiotensin-converting enzymes (ACE) inhibitors, beta-blockers, spironolactone, and several others, are considered cost-effective per the WHO and ACC/AHA definition. Implantable cardiac defibrillators (ICDs) and CardioMEMS™ monitors may be cost-effective under some circumstances [11, 14]; however, analyses of continuous flow left ventricular assist devices (LVADs) showed that they are not cost-effective [15].

Cost-effectiveness analysis is typically based on the outcomes of clinical trials, which are believed to give the best estimates of effectiveness. For example, in the PARADIGM-HF (Prospective Comparison of ARNI with ACEI to Determine Impact on Global Mortality and Morbidity in Heart Failure) study [16], there was a statistically significant mortality benefit with sacubitril/valsartan versus enalapril at a median follow-up of 27 months. A cost-effectiveness analysis based on PARADIGM-HF [13] showed that the model was very sensitive to the duration of benefit, which would need to be more than 36 months, or 9 months beyond the mean follow-up in the trial, for the cost per QALY to remain < $100,000. However, the overall cost per QALY remained well below the threshold of 3× GDP per capita per QALY, even when limited to the proven duration of benefit in the trial. When sacubitril/valsartan was introduced to the market, cardiology leaders in the Veteran Administration (VA) system were surveyed to determine why this combination was underutilized (VA experience, unpublished). Many providers were concerned about cost, despite the published study concluding cost-effectiveness.

From the patient perspective, the concept of value incorporates the payment of medical insurance premiums and out-of-pocket costs. In an analysis of out-of-pocket costs of sacubitril/valsartan versus other less-expensive HF treatments under Medicare Part D, the sacubitril/valsartan mean monthly cost was $57, but varied substantially during the year [17]. Cost was high while the deductible was met, which was projected to occur in January, then reduced greatly under standard coverage until the benefit exhausted in July, after which it remained substantially elevated until the end of the year. For an angiotensin receptor blocker, the deductible was not met by the end of the year. These cost differences may affect patients’ willingness to pay for a treatment.

Physician recommendations also influence those decisions. In a recent study, patients were asked about their willingness to switch from lisinopril to sacubitril/valsartan under several different scenarios [18]. If their provider had no strong recommendation and if switching medication was cost-neutral, 71% of patients would change. If their provider recommended sacubitril/valsartan and it was $5 more per month, 92% would switch. If the out-of-pocket cost was $100 more per month, only 43% of patients would switch; only 60% of patients with income more than $100,000 per year would switch. However, evidence suggests that cost discussions with patients occur infrequently [19]. In a study of 1755 clinic visits with patients who were diagnosed with breast cancer, depression, or rheumatoid arthritis, only 16–30% of providers discussed out-of-pocket costs, and 22–38% discussed any cost or coverage. Although cost communications may be associated with decreased out-of-pocket expenses [20], talking about cost with patients also resulted in a higher likelihood of medication nonadherence (odds ratio: 2.58; 95% CI: 1.14–5.85) [21]. One could speculate that an understanding of the costs of treatment encouraged further cost-saving efforts.

From the payer perspective, Medicare has implemented the Quality Payment Program consisting of 2 payment tracks, one of which is the Merit-Based Incentive Payment System [22]. Quality metrics make up 45% and cost 15% of the provider score, but the balance will shift in 2020 and 2021 with cost metrics increasing and quality metrics decreasing [23]. Performance-linked reimbursement is viewed as a positive way to reconcile the high pricing of new medications; if outcomes from treatment are inconsistent with expectations, the hospital or health system receives a rebate [24]. This has been implemented for HF admission reductions with sacubitril/valsartan and for hypercholesterolemia reductions with PCSK9 inhibitors (full refunds in patients who have had a myocardial infarction or stroke) [25]. Despite this financial adjudication, it is not yet clear whether this saves a health system money, but it likely reduces some of the uncertainty in cost.

Thus, there is no single number or metric that can be imposed to capture all perspectives on value. Cost-effectiveness based on QALY has been a standard measure for nearly all HF therapies but has challenges in calculation and application. For patients, out-of-pocket costs are a highly relevant consideration.

## Remote medicine in heart failure: impact on outcomes and estimated costs

### Larry A. Allen

**Fig. 4:**
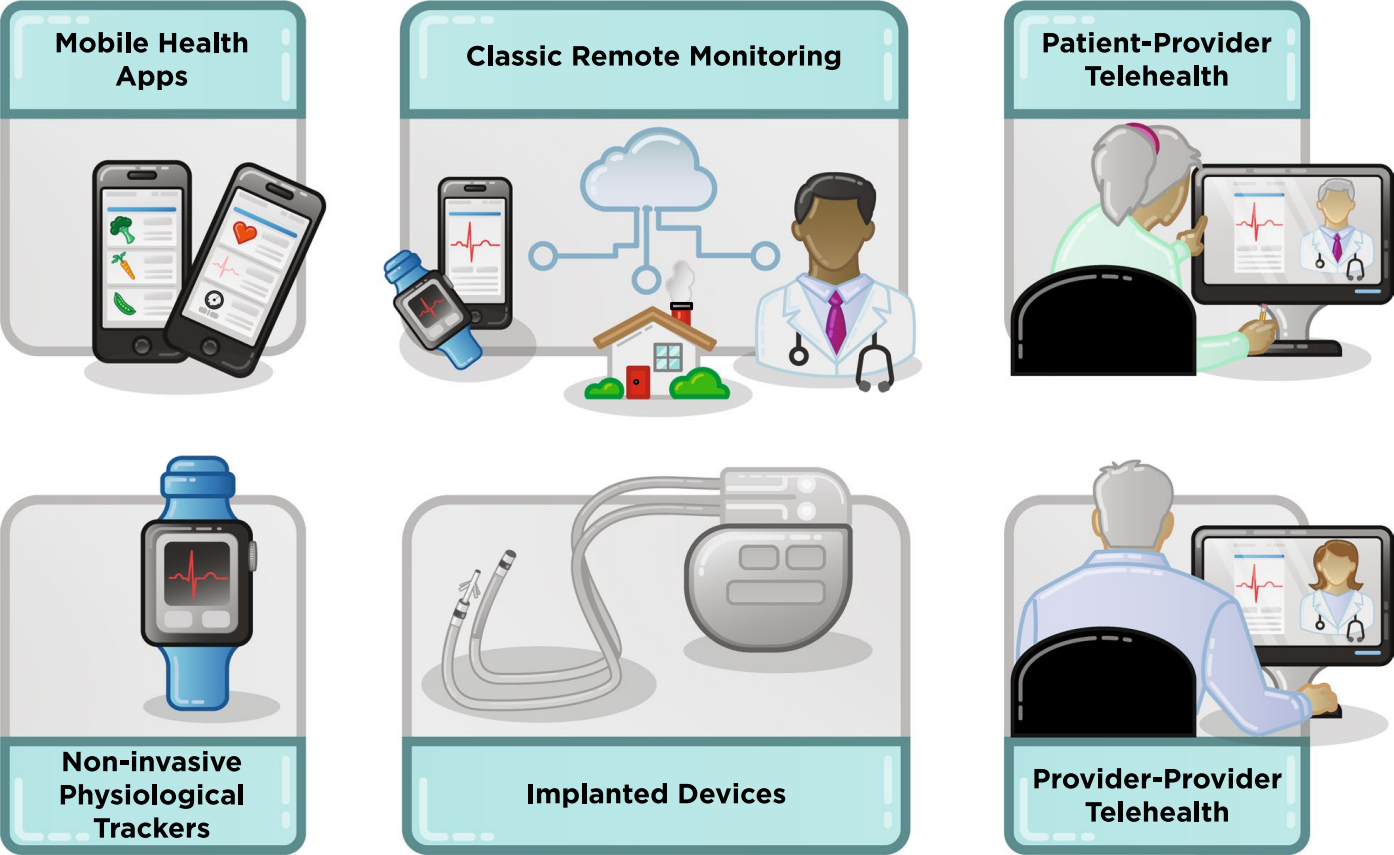
Examples of remote medicine

Traditionally, health care providers have been paid to deliver care based on face-to-face clinical encounters. However, remote “virtual” medicine—also referred to as electronically facilitated care, or “eHealth”—is a rapidly emerging method of delivering health care. Remote patient encounters are paving the way for an expansion of virtual care, commonly referred to as “telehealth.” The use of mobile medical devices and technology to gather patient-generated health data and send them to health care professionals is “remote monitoring.” Health services and information delivered through mobile communication devices, such as smart phones, tablet computers, and monitoring devices, is termed “mHealth” or “mobile health.” Within those platforms are mobile medical applications, software that can be run on a handheld, commercially available, “off-the-shelf” computing platform that meets the definition of a device as defined in section 201(h) of the Federal Food, Drug, and Cosmetic Act. Such applications are to be used as an accessory to a regulated medical device or to transform a mobile platform into a regulated medical device. The proliferation of electronic health records liberates health care providers to access and act upon patient health information from anywhere in the world, including provider-to-provider interactions (“eConsult”) and medication-adherence alerts through prescription fill databases. See Fig. 4.

The evolution of health care is occurring in several different domains. Care was provided in person, then more recently at home, and now electronically through mobile health. Care was initially reactive, then expanded to include broad primary and secondary prevention, and now it has the capacity to be anticipatory. Finally, health monitoring, which used to occur only at infrequent in-person visits, has become daily with devices such as CardioMEMS™ and is evolving toward continuous streaming of physiologic data through the Internet.

Health information has proliferated exponentially. The next challenge in digital medicine is data integration and processing, leading to meaningful actions. For remote health monitoring to be useful, it should ideally transmit to an existing integrated health record where the remote data can be easily accessed, integrated with existing health data, and linked to medications and other aspects of patient care. The data in the electronic health record will need to be processed and sorted carefully by computer algorithms and presented in a way that makes sense and allows for quick review. Patient dashboards are ideal conceptually, but existing dashboards require substantial evolution to meet these needs. Triggers or alarms to drive action based on the data will also need to be developed, along with processes and human resources to review and act on the data, which will require changes in clinical practice operations. These changes in patient care will require education and behavioral changes.

**Fig. 5:**
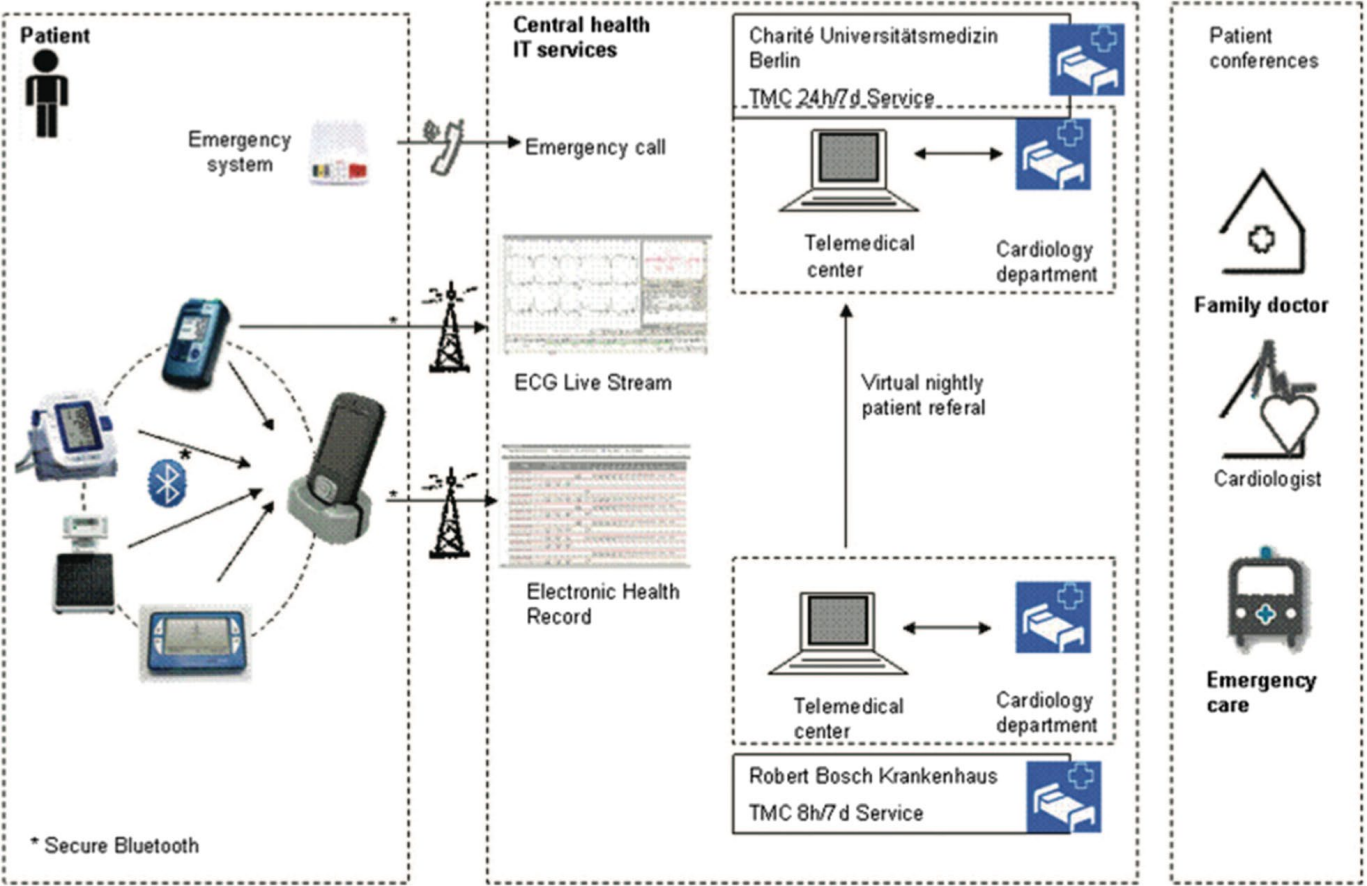
Telehealth example adapted with permission from TIM-HF [27] ECG = electrocardiogram; TMC = telemedical center

Remote interrogation and monitoring of cardiovascular electronic implantable devices (CEIDs) provide a model for basic remote monitoring. The Heart Rhythm Society issued an expert consensus statement [26] showing that remote monitoring of CEIDs reduces the need for in-clinic evaluations, increases early detection of problems, and reduces rates of failed scheduled evaluations. The TIM-HF (Telemedical Interventional Monitoring in Heart Failure) Group algorithm for telemonitoring in HF trials (Fig. 5) [27] illustrates that the situation for the remote monitoring of HF patients is likely to be more complex.

The potential of remote data for diagnosis was illustrated by the Apple Heart Study [28], which enabled enrollment of > 400,000 people in < 8 months. The Apple watch algorithm identified over 2000 patients with an irregular pulse, and 57% of patients who received a notification sought medical attention. Of those with a notification and a subsequent electrocardiogram, 34% had confirmed atrial fibrillation, so the specificity was reasonable. Studies have also shown how automated text messages to patients can change health behaviors for smoking cessation, physical activity, and diet [29].

However, evidence from multiple trials suggests that remote monitoring in chronic HF is more complicated and less likely to improve outcomes without improved approaches [30]. The Diagnostic Outcome Trial in Heart Failure (DOT-HF) study is instructive. Patients with a CEID received an auditory alarm for decreased thoracic impedance, suggesting worsening venous congestion, which was intended to allow time for interventions (e.g., increased diuretics) to avoid hospitalization [31]. Contrary to the intent, hospitalizations and outpatient visits increased when thoracic impedance alerts were provided to patients. Although remote monitoring systems for HF (e.g., daily weight, blood pressure, heart rate, and symptom assessments) have been touted as a way to anticipate clinical worsening and prevent hospitalizations, a series of randomized trials—TIM-HF, Tele-HF (Telemonitoring to Improve Heart Failure Outcomes), and BEAT-HF (Better Effectiveness After Transition—Heart Failure)—all failed to improve patient outcomes [30]. More recently the TIM-HF Group conducted the TIM-HF2 study in a selected HF population, excluding those with major depression based on subgroup analysis of TIM-HF, and using a telemedical center to receive data transmitted daily [32]. This study met its primary endpoint, rekindling interest in this noninvasive remote approach for HF management. Trials such as MultiSENSE (Multisensor Chronic Evaluation in Ambulatory Heart Failure Patients) [33] suggest that more robust CEID-driven data can help inform the care of patients with early worsening HF.

With increasing eHealth and the subsequent proliferation of data and options, there is growing concern that patients and clinicians will be overwhelmed by “noise,” reducing high-value care. Again, DOT-HF showed that more information is not always better. An analysis of virtual visits in the University of Wisconsin Health System found that the introduction of virtual visits triggered more, not less, in-person return visits, and physicians accepted fewer new patients [34].

Reimbursement for digital medicine may also present challenges. Payers offer varying degrees of telehealth reimbursement, and there is a lack of cohesiveness of policies within and between public and private payers, with varying terms of services covered, requirements and restrictions, and patient responsibility for co-pays [35]. Medicare has explicit criteria defining reimbursable telehealth that have limited its widespread use, with very few changes from 2000 to 2019. Outside of telehealth, Medicare is beginning to cover some virtual check-ins, remote evaluation, and eConsults. CMS funding for Medicaid is more permissive, allowing telehealth as long as the service satisfies the federal requirements of “efficiency, economy, and quality of care” [36]. Challenges notwithstanding, if properly integrated into HF care delivery, remote medicine has the potential to support the achievement of quality, value, and cost objectives for systems participating in the BPCI-Advanced Program. For example, virtual visits may increase patient engagement without increasing costs, and telehealth consultations can support collaboration during the transition between acute and post-acute care delivery. Telehealth may also increase the involvement of the patient caregiver by facilitating one on one communication. Economic value is also likely to drive adoption of new eHealth. The high incremental cost-effectiveness ratio seen with CardioMEMS™ contributed to the failure of the CMS to provide a national coverage determination for CardioMEMS™ [37].

In summary, innovations in remote medicine have the promise to transform health care delivery perhaps as much as any other advances in medicine. However, improvements in data veracity, processing, and integration into clinical care will be required for virtual health to significantly improve patient health and the value of care. Other innovations and factors, such as the need for social distancing during a pandemic, may dramatically shift the landscape for eHealth and finally drive uptake of these new opportunities.

## Conclusions

HF care delivery is rapidly evolving in ways that aim to bring more valuable, efficient, patient-centered care while containing costs. BPCI Advanced is a step in that direction, as is the increasing use of digital technologies in the management of HF patients. Much work remains for healthcare providers and society to bring these aims to fruition.

## Data Availability

Not applicable.

## Abbreviations

ACE: Angiotensin-converting enzymes
ACO: Accountable care organizations
BEAT-HF: Better effectiveness after transition-heart failure
BPCI: Bundled payment for care improvement
CEID: Cardiovbascular electronic implantable devices
CI: Confidence interval
CMS: Center for medicare and medicaid services
DOT-HF: Diagnostic outcome trial in heart failure
GDP: Gross domestic product
HF: Heart failure
ICD: Implantable cardiac defibrillators
ICER: Institute for clinical and economic review
LVAD: Left ventricular assist device
MultiSENSE: Multisensor chronic evaluation in ambulatory heart failure patients
PARADIGM-HF: Prospective comparison of ARNI with ACEI to determine impact on global mortality and morbidity in heart failure
PCSK9: Proprotein convertase subtilisin/kexin type 9
QALY: Quality-adjusted life year
SNF: Skilled nursing facilities
TIM-HF: Telemedical interventional monitoring in heart failure
VA: Veteran administration
WHO: World Health Organization
